# Supplementary material for: A Magnetic Photocatalytic Composite Derived from Waste Rice Noodle and Red Mud
Source: Nanomaterials (Basel). 2024 Dec 31;15(1):51. doi: 10.3390/nano15010051 (PMC11723050; doi:10.3390/nano15010051)
Supplement: Supplementary file 1 [file nanomaterials-15-00051-s001.zip › nanomaterials-3356626-supplementary.pdf]

nanomaterials

*Supplementary Information*

# A Magnetic Photocatalytic Composite Derived from Waste Rice Noodle and Red Mud

**Qing Liu, Wanying Ying, Hailing Gou, Minghui Li, Ke Huang, Renyuan Xu, Guanzhi Ding, Pengyu Wang and Shuoping Chen***

1 College of Materials Science and Engineering, Guilin University of technology, Guilin 541004, China; 2120220336@glut.edu.cn (Q.L.); 2120210327@glut.edu.cn (W.Y.); 3212042041650@glut.edu.cn (H.G.); 2120230354@glut.edu.cn (M.L.); 3222042041416@glut.edu.cn (K.H.); 3212042041511@glut.edu.cn (R.X.); 1020220195@glut.edu.cn (G.D.); 2120220368@glut.edu.cn (P.W);

* Correspondence: 2012014@glut.edu.cn

**S1** Synthesis of CQDs solution derived from WRN

The CQDs solution was prepared by hydrothermal treatment of WRN according to our previous work [40-42]. In a typical synthesis, 100 g WRN was ground to a smooth paste in a mortar and mixed with 200 g deionized water. The mixture was heated in a 500 mL sealed Teflon-lined autoclave (Kemi Instrument, Anhui, China) at 200 °C for 10 h. The CQDs solution was collected by filtering as a kind of brown solution.

**S2** General characterization

The characterization of the CQDs/$\gamma$-$Fe_2O_3$ composite material was performed with reference to similar characterization methods as outlined in our prior reports [40-42]. The composition of the main metal ions in the RM leachate was tested using an Agilent 5110 Inductively Coupled Plasma Optical Emission Spectrometer (Agilent, Santa Clara, USA). LC-MS chromatograms of methylene blue after photodegradation were obtained using a Thermo Fisher Ultimate 3000 UHPLC-Q Exactive (Thermo Fisher, Waltham, MA, USA). The particle size distribution of CQDs/$\gamma$-$Fe_2O_3$ composites was measured using a Malvern Mastersizer 3000 (Malvern, Worcestershire, UK). The Raman spectra of the CQDs/$\gamma$-$Fe_2O_3$ composites were recorded using a LabRAM Odyssey Raman Spectrometer (Horiba, Palaiseau, France). The powder X-ray diffraction (PXRD) patterns of CQDs/$\gamma$-$Fe_2O_3$ composite and other control samples were acquired via an X'pert PRO X-ray diffractometer (Panalytical, Malvern, Worcestershire, UK) employing Cu K$\alpha$ radiation ($\lambda$ = 0.15418 Å) at 40 kV and 40 mA, with a scanning speed set at 5° per minute (2$\theta$). The morphology and EDS elemental analysis underwent characterization using a JEM-2100F field emission transmission electron microscope (TEM) from JEOL (Akishima, Tokyo, Japan) at an accelerating voltage of 200 kV. X-ray photoelectron spectroscopy (XPS) for CQDs/$\gamma$-$Fe_2O_3$ composite and commercial $\gamma$-$Fe_2O_3$ was executed using an ESCALAB 250Xi X-ray photoelectron spectrometer (Thermo Fisher, Waltham, MA, USA), powered by an Al K$\alpha$ X-ray as the excitation source. The obtained infrared (IR) spectra of the resultant products were recorded as KBr pellets over the range of 400 to 4000 $cm^{-1}$, utilizing a Nicolet 5700 FT-IR spectrometer (Thermo Fisher, Waltham, MA, USA) featuring a spectral resolution of 4.00 $cm^{-1}$. The determination of BET-specific surface areas for both the CQDs/$\gamma$-$Fe_2O_3$ composite and commercial $\gamma$-$Fe_2O_3$ was executed using a TriStar II 3020 surface area analyzer (Micromeritics, Atlanta, GA, USA) in a nitrogen environment. The assessment was conducted with an initial temperature set at 150 °C and a heating rate of 10 °C per second. To ascertain the points of zero charge (PZC) for the obtained samples, the following method was employed: A sealed centrifugal tube containing 10 mL of 0.01 mol $L^{-1}$ NaCl solution was employed. The pH of the solution was manipulated to fall between 2 and 11 by incremental additions of HCl or NaOH solutions. Subsequently, 0.05 g of the sample was introduced, and the final pH was gauged after 10 hours of agitation. The PZC was determined at the intersection of the pH final versus pH initial curve for the test sample and the control sample.

The UV-VIS absorption spectra of the result materials were meticulously characterized through employment of a UV3100 UV-VIS-NIR spectrophotometer (Shimadzu, Chiyoda, Tokyo, Japan) operating in the mode of diffuse reflection. For reference, $BaSO_4$ was employed as the benchmark material. Investigation into the photo-luminescence spectra of both the CQDs/γ-$Fe_2O_3$ composite and pure γ-$Fe_2O_3$ was conducted using a Cary Eclipse fluorescence spectrophotometer (Varian, Palo Alto, CA, USA), which employed a xenon lamp as its excitation source. The range spanned from 350 to 650 nm, with an excitation wavelength of 325 nm being applied. Evaluation of the photocurrent response (PCR) and electrochemical impedance spectra (EIS) was achieved through utilization of a CH1690 electrochemical analyzer (Huayan, Beijing, China). This apparatus was operated with a 0.5 V bias voltage while subjecting it to illumination from a 300 W Xe lamp, which acted as the source of light.

We delved into the effects of dynamic species like the superoxide radical ($O_2^{-}$), hydroxyl radical (·OH), and photogenerated hole (h+) within the progression of the photocatalytic reaction. To unravel their roles, diverse scavengers were introduced. Precisely, 2-propanol (IPA), 1,4-benzoquinone (BQ, with a purity of 98%), and ethylenediaminetetraacetic acid disodium salt (EDTA–2Na) were strategically employed to pinpoint ·OH, $O_2^{-}$-, and h+, respectively. Employing a methodology akin to the prior photocatalytic activity assay, we incorporated 1 mmol of the scavenger into the reaction mixture. In tandem, electron spin resonance (ESR) spectroscopy was harnessed to gauge the proficiency of radical generation within the CQDs/γ-$Fe_2O_3$ composite and pure γ-$Fe_2O_3$ under 405 nm visible light irradiation. For this purpose, we employed an EMXplus X-band electron paramagnetic resonance spectrometer (Bruker, Karlsruhe, Baden-Württemberg, Germany), with dimethyl pyridine N-oxide (DMPO) serving as the spin-trapping agent for ESR measurements.

**S3** Measurement of photocatalytic performance

Similar to the method employed in our previous work [40-42], the examination of photocatalytic degradation experiments involving organic pollutants was carried out under the influence of visible light irradiation. To commence, a quantity of 2 g/L of the CQDs/γ-$Fe_2O_3$ composite was uniformly dispersed within an aqueous solution containing organic pollutants at an initial concentration of 20 mg/L. This amalgamation underwent shaking on an OS-20 shaker table (Oaiclab, Zhejiang, China), maintained at 25 °C in darkness, for a duration of 1 hour. Subsequently, the mixture was positioned beneath a 20 W 405 nm purple light lamp emitting light at an intensity of $8\times10^4$ LUX. This purple light lamp emitted wavelengths ranging from 340 to 450 nm (Refer to Figure S5 in ESI). Repetitive experiments were meticulously prepared as individual samples. At designated time intervals, one of the samples was extracted and subsequently subjected to centrifugation. The concentrations of organic pollutants within these samples were quantified using UV-VIS spectrophotometry through utilization of a UV3100 UV-VIS-NIR spectrophotometer (Shimadzu, Chiyoda, Tokyo, Japan). Testing wavelengths were as follows: 664 nm (methylene blue), 618 nm ( malachite green), 582 nm (methyl violet), 543 nm (basic fuchsin), 554 nm (rhodamine B). The extent of photocatalytic degradation pertaining to the organic pollutants was determined by evaluating the $C/C_0$ value, where C represents the concentration of the organic pollutant at a specific time and $C_0$ symbolizes the initial concentration of the organic pollutant. It's important to note that each outcome of photocatalytic degradation reported in this study reflects the average measurement derived from three samples sharing identical compositions.

The photocatalytic kinetics can be elucidated through the Langmuir-Hinshelwood model [71], wherein the integral representation is as follows:

$$t = (\frac{1}{K_r K})\ln(\frac{C_0}{C}) + \frac{C_0 - C}{K_r} \qquad (1)$$

Here, *t* signifies the irradiation time; *K* stands for the equilibrium constant governing the adsorption of the organic pollutant on the catalyst, while $K_r$ reflects the reaction's limiting rate at the utmost coverage within the given circumstances; $C_0$ denotes the initial concentration of the organic pollutant, and *C* represents the pollutant concentration at time t.

When the organic pollutant's initial concentration is low, the second term in equation (1) becomes negligible, and therefore, it can be disregarded. Consequently, the kinetics analysis for photocatalytic degradation in this study employed the subsequent equation:

$$\ln(\frac{C_0}{C}) = K_r K t = K_{app} t \quad (2)$$

Here, $K_{app}$ stands for the apparent degradation rate constant, measured in $min^{-1}$, serving as the fundamental kinetic parameter.

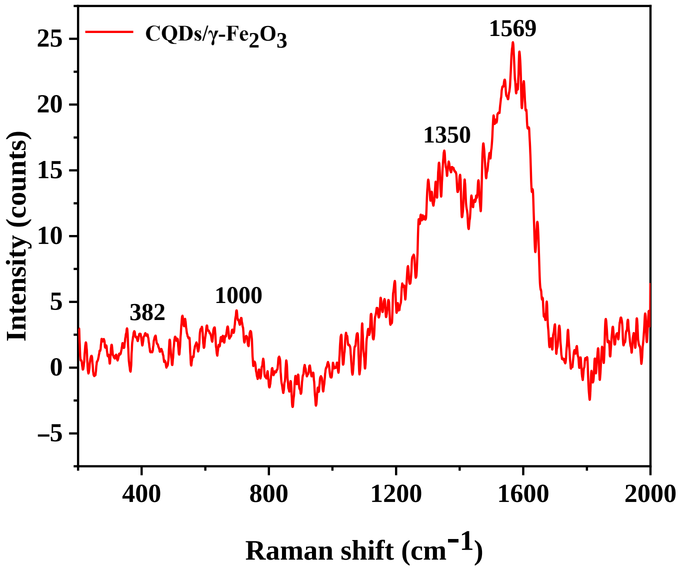


**Figure S1.** The Raman spectrum of CQDs/γ-$Fe_2O_3$ composite (CF-2 sample).

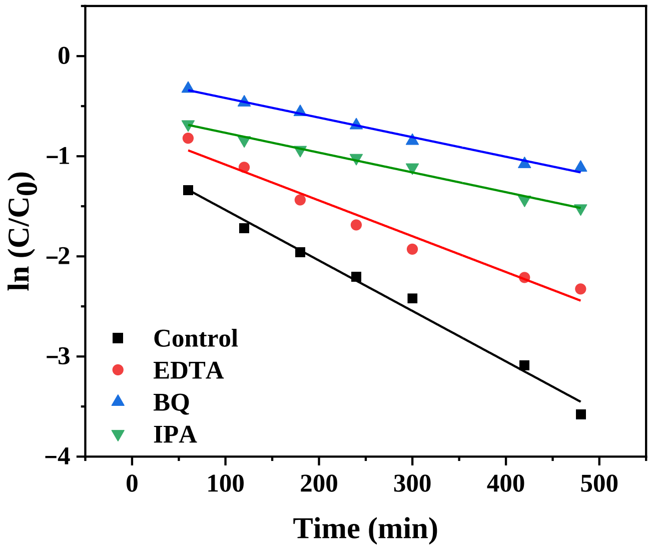


**Figure S2.** The pseudo-first-order kinetic fitting of photocatalytic degradation of CF-2 sample to methylene blue using different scavengers (EDTA-2Na, BQ, IPA).

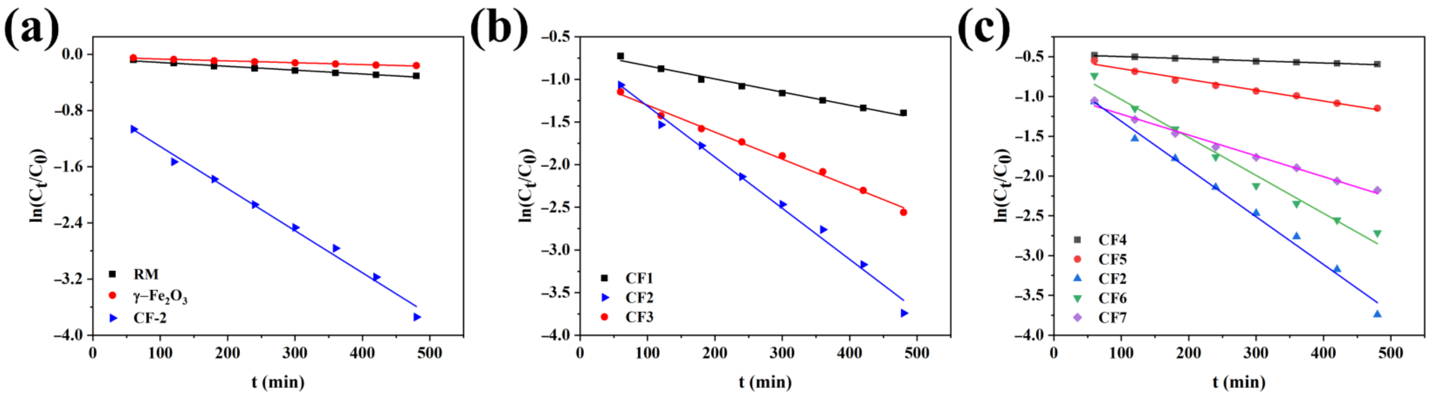


**Figure S3 (a)** The pseudo-first-order kinetic fitting of photocatalytic degradation for CQDs/γ-$Fe_2O_3$ composite (CF-2 sample), commercial γ-$Fe_2O_3$, and unmodified RM. (b) The pseudo-first-order kinetic fitting of photocatalytic degradation for CQDs/γ-$Fe_2O_3$ composites under different pH conditions. (c) The pseudo-first-order kinetic fitting of photocatalytic degradation for CQDs/γ-$Fe_2O_3$ composites using different CQDs dosages.

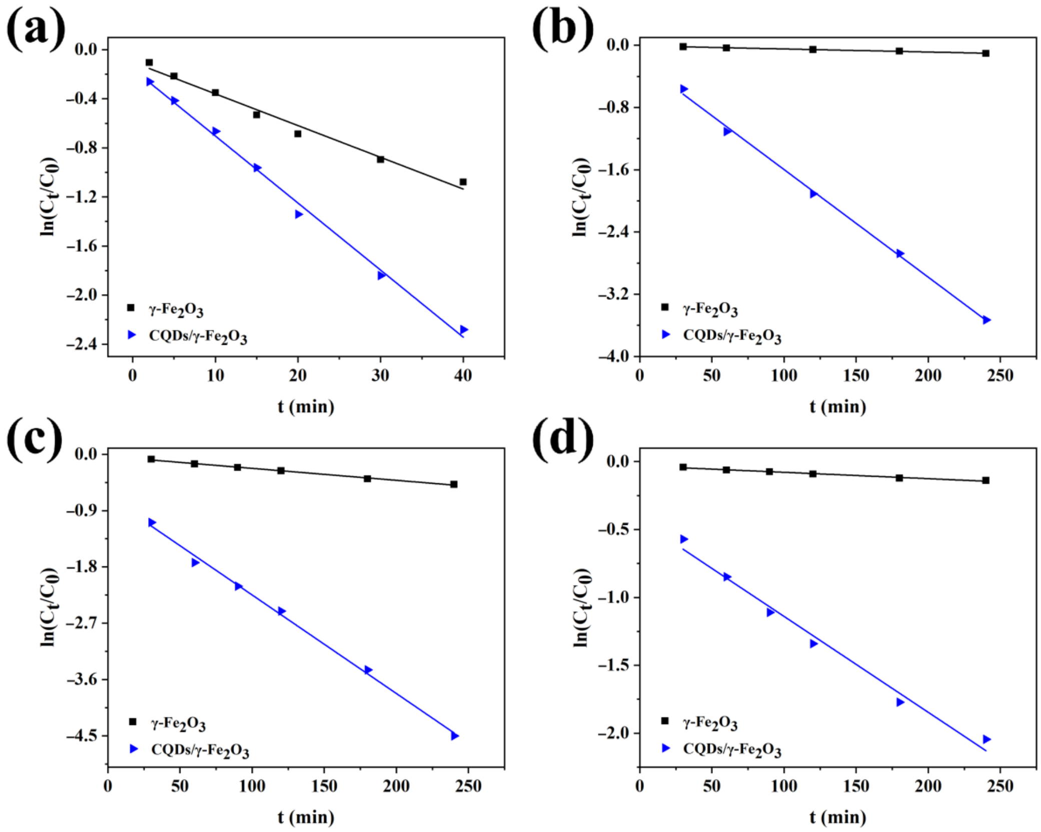


**Figure S4** The pseudo-first-order kinetic fitting of photocatalytic degradation for the CQDs/γ-$Fe_2O_3$ composite (CF-2 sample) and commercial γ-$Fe_2O_3$ to malachite green (a), methyl violet (b), basic fuchsin (c), and rhodamine B (d).

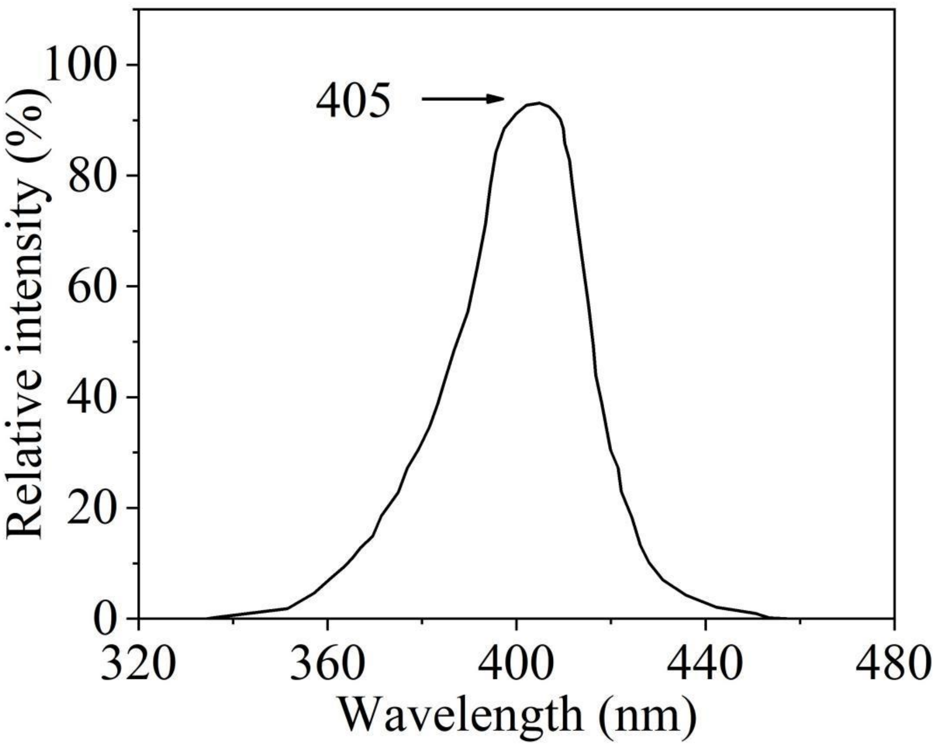


**Figure S5** The emission spectrum of the 405 nm purple light lamp.

**Table S1.** The kinetic parameters of photocatalytic degradation for CQDs/$\gamma$-$Fe_2O_3$ and other control samples to methylene blue

| **Photocatalyst** | $K_{app}$ | $R^2$ |
|---|---|---|
| CF-1 | $0.00154 \pm 8.24963 \times 10^5$ | 0.98309 |
| CF-2 | $0.00599 \pm 2.36378 \times 10^4$ | 0.99076 |
| CF-3 | $0.00316 \pm 1.12763 \times 10^4$ | 0.99244 |
| CF-4 | $2.68119 \times 10^4 \pm 1.29201 \times 10^5$ | 0.98626 |
| CF-5 | $0.00136 \pm 6.98988 \times 10^5$ | 0.98436 |
| CF-6 | $0.00477 \pm 2.46879 \times 10^4$ | 0.98415 |
| CF-7 | $0.00262 \pm 1.01794 \times 10^4$ | 0.99100 |
| $\gamma$-$Fe_2O_3$ | $2.62183 \times 10^4 \pm 1.12989 \times 10^5$ | 0.98898 |
| RM | $5.45815 \times 10^4 \pm 2.8176 \times 10^5$ | 0.98426 |

**Table S2.** The kinetic parameters of photocatalytic degradation for composite (CF-2 sample), commercial $\gamma$-$Fe_2O_3$ to malachite green, methyl violet, basic fuchsin, and rhodamine B.

| Photocatalyst | dye | $K_{app}$ | $R^2$ |
|---|---|---|---|
| $\gamma$-$Fe_2O_3$ | malachite green | $0.02586 \pm 0.00149$ | 0.98362 |
| CQDs/$\gamma$-$Fe_2O_3$ | | $0.05465 \pm 0.0017$ | 0.9952 |
| $\gamma$-$Fe_2O_3$ | methyl violet | $3.93215\times10^{4} \pm 1.41639\times10^{5}$ | 0.99612 |
| CQDs/$\gamma$-$Fe_2O_3$ | | $0.01383 \pm 3.52318\times10^{4}$ | 0.99806 |
| $\gamma$-$Fe_2O_3$ | basic fuchsin | $0.00191 \pm 6.72391\times10^{5}$ | 0.99508 |
| CQDs/$\gamma$-$Fe_2O_3$ | | $0.01575 \pm 4.5675\times10^{4}$ | 0.99665 |
| $\gamma$-$Fe_2O_3$ | rhodamine B | $4.69147\times10^{4} \pm 2.81471\times10^{5}$ | 0.98581 |
| CQDs/$\gamma$-$Fe_2O_3$ | | $0.00707 \pm 4.28297\times10^{4}$ | 0.98552 |

## References

[40]. Jin, X.; Che, R.; Yang, J.; Liu, Y.; Chen, X.; Jiang, Y.; Liang, J.; Chen, S.; Su, H. Activated Carbon and Carbon Quantum Dots/Titanium Dioxide Composite Based on Waste Rice Noodles: Simultaneous Synthesis and Application in Water Pollution Control. *Nanomaterials.* **2022**, *12*, 472.

[41]. Jin, X.Y.; Ying, W.Y.; Che, R.J.; Xiao, P.; Zhou, Y.Q.; Liu, Y.; Liu, M.Y.; Chen, S.P. CQDs/ZnO composites based on waste rice noodles: preparation and photocatalytic capability†. *RSC Adv.* **2022**, *12*, 23692-23703.

[42]. Ying, W.Y.; Liu, Q.; Jin, X.Y.; Ding, G.Z.; Liu, M.Y.; Wang, P.Y.; Chen, S.P. Magnetic carbon quantum dots/iron oxide composite based on waste rice noodle and iron oxide scale: preparation and photocatalytic capability. *Nanomaterials*. **2023**, *13(18)*, 2506.

[71]. Kumara, K.V.; Porkodi, K.; Rocha, F. Langmuir–Hinshelwood kinetics—A theoretical study. *Catal. Commun.* 2008, 9, 82–84.
